# Serological Detection of O‐Type and A‐Type Antibodies Against Foot‐and‐Mouth Disease in Tongliao City, 2021–2022

**DOI:** 10.1155/vmi/9983736

**Published:** 2026-08-27

**Authors:** Rong Zhao, Xiong Dai, Jun Ai, Tianlong Hu, Ruiying Wang, Yanlin Li, Jige Xin, Diangang Han

**Affiliations:** ^1^ Yunnan Key Laboratory of Veterinary Etiological Biology, College of Veterinary Medicine, Yunnan Agricultural University, Kunming 650201, China, ynau.edu.cn; ^2^ Animal Quarantine Laboratory, Technology Center of Kunming Customs, Kunming 650200, China

**Keywords:** foot-and-mouth disease, foot-and-mouth disease virus, serological detection, Serotypes O and A

## Abstract

Foot‐and‐mouth disease (FMD) is an acute, febrile, and highly contagious viral disease caused by the foot‐and‐mouth disease virus (FMDV). Serological testing for FMDV antibodies is an effective method to assess the immune response following vaccination. In this study, 36,510 bovine blood samples were collected from both households and farms across 8 districts of Tongliao City, Inner Mongolia Autonomous Region, China, between January 2021 and December 2022. Enzyme‐linked immunosorbent assay (ELISA) was employed to detect serum antibodies. The results indicated that the seropositivity rates of O‐type FMDV antibodies in eight regions (A–H) of Tongliao City during 2021–2022 ranged from 73.09% to 100% in 2021 and 83.75%–100% in 2022, respectively. Significant differences were observed among the regions. In 2021, the immune protection levels of A‐type FMDV antibodies in Regions B and G were below 70%, at 65.38% and 68.40%, respectively. In 2021, the positive rates of FMDV O‐type and A‐type antibodies were significantly higher (*p* < 0.05) on farms compared to households, indicating a higher trend in farms. However, in 2022, no significant difference was observed between farms and households (*p* > 0.05). Seasonally, the positive rates of O‐type and A‐type FMDV antibodies in spring, summer, autumn, and winter of 2021 were 80.81%–97.67% and 78.27%–94.07%, respectively, while in 2022, they were 83.18%–96.50% and 80.14%–94.76%, respectively. During the period from 2021 to 2022, FMD immunization efforts in various regions of Tongliao City achieved generally satisfactory immunization outcomes. However, Regions B and G require enhanced immunization and reevaluation. This information serves as a valuable reference for the formulation of prevention and control strategies for FMD Serotypes O and A.

## 1. Introduction

Foot‐and‐mouth disease (FMD) is a highly contagious viral infection caused by the foot‐and‐mouth disease virus (FMDV), primarily affecting cloven‐hoofed animals such as pigs, cattle, and sheep [1]. FMD ranks among the most detrimental infectious diseases in the livestock industry, leading to substantial economic losses [2]. The virus spreads through various bodily excretions and secretions, including respiratory droplets, milk, and semen, as well as via aerosols, which facilitates its rapid transmission [3]. FMDV is a nonenveloped, single‐stranded RNA virus with seven distinct serotypes: A, O, C, Asia1, SAT1, SAT2, and SAT3. Cross‐immune protection does not exist between these serotypes [4, 5], necessitating serotype‐specific vaccines for effective immunization. Different FMDV serotypes exhibit a recurring tendency within a geographical area, characterized by periodic emergence and transmission. Currently, FMDV Serotypes O and A are the predominant and most detrimental strains impacting the livestock industry in China and neighboring countries [6, 7]. FMDV Serotype C has not been isolated by the World Organization for Animal Health (WOAH) and Food and Agriculture Organization (FAO) Reference Laboratory network since 2004, and no Serotype C outbreaks have been reported to WOAH since that time. The remaining serotypes continue to circulate in various countries and territories around the world (https://www.woah.org/en/disease/foot-and-mouth-disease/, accessed on February 28, 2025).

Clinically, FMD manifests with symptoms such as fever, anorexia, oral mucosal ulcers and hemorrhages, vesicles and lesions on hooves, and occasional skin blisters and plaques on udders [8]. The disease spreads rapidly, exhibits high morbidity, lacks seasonal patterns, and often results in sudden death and high mortality rates in young animals. WOAH has designated it as a notifiable animal disease. FMD is a highly contagious transboundary animal disease (TAD) that has caused significant losses to the livestock industry and economic development worldwide. The UK experienced an FMD epidemic in 1967, which led to the culling of approximately 500,000 animals [9]. In 1997, Taiwan experienced a devastating outbreak of FMD that spread among pig herds, resulting in the culling of nearly 4 million pigs [10]. Between 2007 and 2012 in Ethiopia, FMD virus Serotypes O, A, SAT2, and SAT1 were identified as the causal serotypes, whereas O was the dominant serotype and SAT2 was the serotype that showed an increase in relative frequency of occurrence [11]. During the 2010 outbreak of FMD in Japan, approximately 290,000 animals were culled [12]. To date, several regions remain under significant threat from this disease. Nigeria, a large and densely populated country in West Africa, continues to experience the circulation of FMDV Serotypes A, O, SAT1, and SAT2 as recently as 2020 [13–17]. Given that Nigeria’s demand for animal products significantly exceeds its domestic production capacity, the importation of livestock and poultry from neighboring countries has substantially increased the risk of introducing FMDV [18]. FMD is endemic in Thailand. To effectively prevent and control the disease, the Thai government has implemented a biannual routine vaccination program for ruminants such as cattle and sheep. However, despite these efforts, FMD outbreaks continue to be reported annually in several regions of the country [19].

Since the discovery of FMD, it has spread extensively across Asia, Africa, the Middle East, and Latin America, with its origins traced to the Arab region of the Middle East. China stands out as one of the most severely affected countries by FMD, where the disease has significantly impacted the country’s livestock industry. The first recorded outbreak of FMD in China occurred in 1958, primarily affecting the Xinjiang Uyghur Autonomous Region and Yunnan Province [20]. FMD outbreaks have occurred in several provinces in China, primarily involving Serotypes O and A. To combat the spread of FMD, China has implemented a series of protective measures, with mandatory vaccination being a key strategy. The efficacy of vaccine immunization is affected by a variety of factors, including vaccine quality, transportation conditions, and immunization techniques, which necessitates regular evaluation of vaccine performance in FMD prevention and control. Small‐scale farming and free‐range grazing are recognized as major risk factors for elevated FMD prevalence in China. Additionally, multiple factors, including climate conditions, livestock reproductive status, animal density, and interprovincial animal transportation, play a critical role in the transmission of FMD [21]. In endemic areas, FMD is managed through clinical diagnosis, laboratory testing, and routine mass vaccination [22]. In FMD‐free countries, preventive strategies focus on reducing infection sources, protecting susceptible animals, and restricting the movement of animals and their products [23].

Serum antibody monitoring is essential for evaluating the immune response to FMD postvaccination. The “National Compulsory Immunization Program for Animal Diseases in 2021,” the “National Compulsory Immunization Program for Animal Diseases in 2022,” and the “National Guidelines on Compulsory Immunization against Animal Diseases (2022–2025)” in China mandate that the serological antibody qualification rate for FMDV should be no less than 70%. The results of such monitoring are frequently utilized to gauge the animals’ resistance to pathogens, enabling the formulation of targeted prevention and control measures based on immune status, as well as the adjustment and optimization of immunization programs to ensure the health and well‐being of livestock and poultry [24]. Due to its high efficiency and low cost, and its rapid and cost‐effective characteristics, serum antibody detection is a widely utilized diagnostic assay in practical applications. The sensitivity of this method is significantly influenced by the timing of viral exposure, which directly affects antibody levels [25]. Jeong et al. immunized cattle with FMDV to construct a phage‐displayed single‐chain variable fragment (scFv) library and screened for inactivated FMDV Serotypes O and A. They adopted indirect enzyme‐linked immunosorbent assay (iELISA) to identify scFvs with high‐affinity serotype‐specific antibodies and pan‐serotype‐specific antibodies, which were then tested using a competitive ELISA (cELISA). It was found that the foot‐and‐mouth antiviral O‐type‐specific antibody BvO17 could successfully detect the serum samples of vaccinated cattle and virus‐challenged pigs. The sensitivity even exceeded that of the commercial FMDV O‐type antibody detection kit based on mouse monoclonal antibodies (mAbs). Therefore, in cELISA applications, natural host antibodies can replace mouse mAbs [26]. Kashem et al. established serotype‐specific mAbs‐based blocking ELISA (mAb‐bELISAs), and developed four different mAb‐bELISAs for the detection of antibodies against FMDV O, A, Asia1, and SAT2. When the threshold was set at 50%, the diagnostic specificity of the four mAb‐bELISAs showed 99.74%, 98.01%, 96.59%, and 98.55%, respectively, with corresponding diagnostic sensitivity (DSe) values of 98.93%, 98.25%, 100%, and 87.50%. Moreover, when the cutoff value was 30%, the O‐type and SAT2‐specific mAb‐bELISAs of swine serum exhibited a DSe of 100%. All the developed mAb‐bELISAs demonstrated high reproducibility and repeatability without cross‐reactivity [27]. Tian et al. developed an iELISA assay based on the p34 protein for detecting antibodies against African swine fever virus (ASFV), with positive serological results indicating ongoing or past ASFV infection [28]. Kweon et al. employed two serological assays to analyze the kinetic profile of antibodies against severe acute respiratory syndrome coronavirus 2 (SARS‐CoV‐2) in COVID‐19 patients [29]. Consequently, serological tests are commonly used for detecting viral antibodies.

Inner Mongolia boasts the natural advantages of extensive grasslands and superior livestock and poultry breeds, making cattle breeding a prominent feature of the region. Tongliao City, situated in southeastern Inner Mongolia, is a key area for cattle breeding. Its strategic location and well‐developed transportation network facilitate active trade in livestock and poultry products, thereby elevating the risk of FMD transmission and increasing the complexity of disease prevention and control. To address this issue, this study collected a total of 36,510 blood samples from cattle in Tongliao City in 2021 and 2022. The primary objectives were to assess FMD Type O and A antibody levels, evaluate vaccination efficacy, and provide a scientific basis for formulating comprehensive FMD prevention and control strategies.

## 2. Materials and Methods

### 2.1. Sample Collection

A large‐scale cross‐sectional observational study was carried out. From 2021 to 2022, a total of 36,510 cattle blood samples were collected from both households and farms across eight regions of Tongliao City, Inner Mongolia. These regions, labeled A to H, include Zuoyi Zhongqi, Horqin, Zuoyi Houqi, Kulun, Naiman, Kailu, Zhaluotai, and Hulinnaole (Table 1). The samples were collected from various regions and seasons, resulting in a sampling ratio of over 0.5%, which satisfies the criteria for large‐scale statistical analysis. Blood samples were collected via the jugular vein, with 2–3 mL of each sample being drawn. The samples were placed at room temperature for 1–2 h, followed by serum separation via centrifugation at 3000 rpm for 15 min. The separated serum was subsequently transferred to Eppendorf tubes for subsequent ELISA testing.

**TABLE 1:** Bovine serum collection records in eight regions of Tongliao City.

Region	Source	2021				2022				Total
		Spring	Summer	Autumn	Winter	Spring	Summer	Autumn	Winter	
A	Household	7	471	461	216	143	303	46	137	1784
	Farm	5	624	91	437	176	80	121	57	1591

B	Household	434	209	1017	474	0	65	88	142	2429
	Farm	378	0	986	227	37	1611	1118	729	5086

C	Household	94	152	751	1013	597	552	1156	245	4560
	Farm	0	517	0	135	777	620	833	188	3070

D	Household	0	51	439	176	161	70	31	12	940
	Farm	0	3155	2472	1041	162	418	188	150	7586

E	Household	121	81	0	0	0	0	100	0	302
	Farm	0	0	0	0	0	0	0	0	0

F	Household	354	523	922	365	636	2409	648	221	6078
	Farm	242	282	0	231	0	93	339	0	1187

G	Household	0	0	0	705	0	0	0	0	705
	Farm	0	0	0	514	96	80	150	179	1019

H	Household	84	9	0	0	30	0	0	0	123
	Farm	0	0	0	0	50	0	0	0	50

Total		1719	6074	7139	5534	2865	6301	4818	2060	36,510

### 2.2. Main Reagents

Improved FMD O‐type antibody detection liquid‐phase blocking ELISA kit (purchased from Lanzhou Veterinary Research Institute, Chinese Academy of Agricultural Sciences; batch number: 20210519101‐1) and improved FMD A‐type antibody detection liquid‐phase blocking ELISA kit (purchased from Lanzhou Veterinary Research Institute, Chinese Academy of Agricultural Sciences; batch number: 20210423103‐5) were used. These kits have been officially approved by the Ministry of Agriculture and Rural Affairs of China.

### 2.3. Test Procedure

ELISA was employed to detect O‐type and A‐type antibodies against FMD. The procedure was conducted according to the instructions provided with the enhanced ELISA kit from the Lanzhou Veterinary Research Institute, Chinese Academy of Agricultural Sciences.

### 2.4. Interpretation

Set up four viral antigen control wells in a 96‐well plate, and the OD_450 nm_ value of the viral antigen control should be above 1.0. The titer of the positive control antibody should be within 1:1024 ± 1, and the titer of the negative control serum antibody should be less than 1:8. For the viral antigen control wells, discard the highest and lowest OD_450nm_ values, calculate the average of the remaining two values, and then divide by 2 to obtain the cutoff value. If the cutoff value is greater than or equal to the tested serum value, the result is positive; if the cutoff value is less than the tested serum value, the result is negative.

### 2.5. Data Analysis

The test data were compiled and analyzed using Excel 2016 software, and SPSS 23.0 was used to analyze the significance of the antibody‐positive rates via Pearson’s chi‐squared test, and multiple comparisons were conducted by chi‐square multiple comparison using *Z* test. Statistical significance was defined as a probability value (P) less than the significance level of 5% (*p* < 0.05).

## 3. Results

### 3.1. The FMDV Antibody‐Positive Rates Across From Different Regions

A total of 36,510 serum samples were collected from eight distinct regions of Tongliao City. The bovine serum samples collected during 2021–2022 were tested for FMDV O‐type and A‐type antibodies using enhanced liquid‐phase blocking ELISA kits. The test results are presented in Table 2. Apart from the positive rates of FMDV A‐type antibodies in Regions B and G in 2021, which were 68.40% and 65.38%, respectively, failing to meet the national requirement of ≥ 70%, the positive rates of both O‐type and A‐type FMDV antibodies across all eight regions (A–H) of Tongliao City in 2021–2022 exceeded 70%. Notably, for A‐type, the highest rate in 2021 was 100% in Region H, while the lowest was 65.38% in Region G. In 2022, Region E recorded the highest rate at 100%, whereas Region H had the lowest at 83.75%. Significant differences were observed among the regions (Table 2). Consequently, enhanced immunization and surveillance measures will be implemented in Regions B and G.

**TABLE 2:** Positive rate of FMDV O‐type and A‐type antibody in different regions.

Year	2021					2022				
Regions	Total	Positive samples of O‐type antibody	Positive rate of O‐type antibody	Positive samples of A‐type antibody	Positive rate of A‐type antibody	Total	Positive samples of O‐type antibody	Positive rate of O‐type antibody	Positive samples of A‐type antibody	Positive rate of A‐type antibody
A	2312	2072	89.62%^a^	2125	91.91%^a^	1063	979	92.10%^abc^	947	89.09%^ab^
B	3725	2831	76.00%^b^	2548	68.40%^b^	3790	3681	97.12%^d^	3664	96.68%^c^
C	2662	2136	80.24%^c^	2186	82.12%^c^	4968	4415	88.87%^ce^	4249	85.53%^b^
D	7334	6946	94.71%^d^	6818	92.96%^a^	1192	1044	87.58%^e^	1066	89.43%^a^
E	202	197	97.52%^d^	196	97.03%^a^	100	100	100.00%^bd^	100	100.00%^c^
F	2919	2747	94.11%^d^	2688	92.09%^a^	4346	3999	92.02%^ab^	3754	86.38%^ab^
G	1219	891	73.09%^b^	797	65.38%^b^	505	481	95.25%^bd^	448	88.71%^ab^
H	93	93	100.00%^d^	93	100.00%^a^	80	67	83.75%^ace^	73	91.25%^abc^

*Note:* Data in the same column followed by the same superscripts indicate no significant difference (*p* > 0.05), while different lowercase letters denote significant differences at the 0.05 level. This convention applies to the following tables as well.

### 3.2. The FMDV Antibody‐Positive Rates Across From Different Sources

The serum samples in this study were collected from both households and farms, with the statistical results for different sources presented in Table 3. During 2021–2022, the positive rate of samples from different sources exceeded 80%. Specifically, in 2021, the positive rates of FMDV O‐type and A‐type antibodies were significantly higher (*p* < 0.05) on farms compared to households, indicating a trend of higher positivity in farms. However, in 2022, no significant difference was observed between farms and households (*p* > 0.05) (Table 3).

**TABLE 3:** Positive rate of FMDV O‐type and A‐type antibody in different sources.

Year	2021					2022				
Sources	Total	Positive samples of O‐type antibody	Positive rate of O‐type antibody	Positive samples of A‐type antibody	Positive rate of A‐type antibody	Total	Positive samples of O‐type antibody	Positive rate of O‐type antibody	Positive samples of A‐type antibody	Positive rate of A‐type antibody
Households	9129	7866	86.16%^a^	7637	83.66%^a^	7792	7184	92.20%^a^	6946	89.14%^a^
Farms	11,337	10,047	88.62%^b^	9814	86.57%^b^	8252	7582	91.88%^a^	7355	89.13%^a^

*Note:* Data in the same column followed by the same superscripts indicate no significant difference (*p* > 0.05), while different lowercase letters denote significant differences at the 0.05 level.

### 3.3. The FMDV Antibody‐Positive Rates Across From Different Seasons

The results of serum antibody detection are presented in Table 4. The positive rates of O‐type and A‐type antibodies from 2021 to 2022 exceeded 70%, with the lowest rate being 78.27% and the highest rate being 97.67%. These positive rates meet the requirement of being greater than or equal to 70%, as stipulated in the 2020–2022 National Compulsory Immunization Program for Zoonotic Infectious Diseases. In 2021, the positive rates of O‐type and A‐type antibodies were highest in spring, at 97.67% and 94.07%, respectively, which are significantly higher than other seasons (*p* < 0.05). Meanwhile, the positive rates of O‐type and A‐type antibodies were lowest in autumn (*p* < 0.05). In 2022, the positive rates of O‐type and A‐type antibodies were highest in winter, at 96.50% and 94.76%, respectively, and lowest in spring, at 83.18% and 80.14%, respectively (Table 4).

**TABLE 4:** Positive rate of FMDV O‐type and A‐type antibody in different seasons.

Year	2021					2022				
Seasons	Total	Positive samples of O‐type antibody	Positive rate of O‐type antibody	Positive samples of A‐type antibody	Positive rate of A‐type antibody	Total	Positive samples of O‐type antibody	Positive rate of O‐type antibody	Positive samples of A‐type antibody	Positive rate of A‐type antibody
Spring	1719	1679	97.67%^a^	1617	94.07%^a^	2865	2383	83.18%^a^	2296	80.14%^a^
Summer	6074	5643	92.90%^d^	5484	90.29%^d^	6301	5776	91.67%^c^	5684	90.21%^c^
Autumn	7139	5769	80.81%^c^	5588	78.27%^c^	4818	4619	95.87%^b^	4369	90.68%^c^
Winter	5534	4822	87.13%^b^	4762	86.05%^b^	2060	1988	96.50%^b^	1952	94.76%^b^

*Note:* Data in the same column followed by the same superscripts indicate no significant difference (*p* > 0.05), while different lowercase letters denote significant differences at the 0.05 level.

### 3.4. The FMDV Antibody‐Positive Rates Across Different Seasons and Sources

The results of serum antibody detection from different seasons and sources are presented in Table 5. The positive rates of FMDV antibodies in spring, summer, autumn, and winter of 2021–2022 were all above 80%. Specifically, the positive rates of FMDV O‐type and A‐type antibodies in households were lower in autumn and winter, at 88.80% and 84.70%, and 84.13% and 82.65% in winter, respectively. For households, the positive rates in summer were significantly higher than those in winter (*p* < 0.05). Differently, the positive rates of FMDV O‐type and A‐type antibodies in farms were lower in spring and autumn, at 84.61% and 82.16% in spring, and 85.15% and 81.99% in autumn, respectively. For farms, the positive rates in winter were significantly higher than those in summer (*p* < 0.05) (Table 5).

**TABLE 5:** Positive rate of FMDV O‐type and A‐type antibody in different seasons and sources.

Sources	Households					Farms				
Seasons	Total	Positive samples of O‐type antibody	Positive rate of O‐type antibody	Positive samples of A‐type antibody	Positive rate of A‐type antibody	Total	Positive samples of O‐type antibody	Positive rate of O‐type antibody	Positive samples of A‐type antibody	Positive rate of A‐type antibody
Spring	2661	2435	91.51%^a^	2333	87.67%^a^	1923	1627	84.61%^a^	1580	82.16%^a^
Summer	4895	4472	91.36%^a^	4394	89.77%^c^	7480	6947	92.87%^c^	6774	90.56%^c^
Autumn	5659	5025	88.80%^c^	4793	84.70%^b^	6298	5363	85.15%^a^	5164	81.99%^a^
Winter	3706	3118	84.13%^b^	3063	82.65%^b^	3888	3692	94.96%^b^	3651	93.90%^b^

*Note:* Data in the same column followed by the same superscripts indicate no significant difference (*p* > 0.05), while different lowercase letters denote significant differences at the 0.05 level.

### 3.5. Annual FMDV Antibody‐Positive Rates

A total of 36,510 serum samples were collected from 2021 to 2022. The positive rates of FMDV O‐type antibodies were 87.53% and 92.03% for 2021 and 2022, respectively, with significant differences between the two years (*p* < 0.05). Similarly, the positive rate of FMD A‐type antibodies was 85.27% in 2021 and 89.14% in 2022, also with statistically significant annual differences (*p* < 0.05). In both 2021 and 2022, both the positive rates of FMD O‐type and A‐type antibodies were all above the national qualifying immunization antibody threshold of 70% (Table 6).

**TABLE 6:** The results of FMDV antibody‐positive rates analyzed from years.

Year	Total	Positive samples of O‐type antibody	Positive rate of O‐type antibody	Positive samples of A‐type antibody	Positive rate of A‐type antibody
2021	20,466	17,913	87.53%^a^	17,451	85.27%^a^
2022	16,044	14,766	92.03%^b^	14,301	89.14%^b^

*Note:* Data in the same column followed by the same superscripts indicate no significant difference (*p* > 0.05), while different lowercase letters denote significant differences at the 0.05 level.

## 4. Discussion

In recent years, the rapid economic growth has led to an increase in the cross‐regional movement of people, material transportation, and livestock migration, significantly complicating the prevention and control of major animal diseases. Currently, no targeted and effective treatments for FMD have been developed, making vaccine immunization the primary strategy for controlling FMD in endemic areas [30]. However, the practical application of vaccines faces several significant challenges. The vaccine production process involves the cultivation of FMDV. While manufacturers adhere to rigorous safety standards and procedures, there remains a theoretical possibility of accidental virus release, which could pose a potential threat to the surrounding environment. Additionally, trace amounts of nonstructural proteins (NSPs) of the FMD virus may persist during vaccine preparation, leading to interference with serological identification methods that rely on detecting NSPs in infected animals, potentially compromising the accuracy of these tests. Lastly, even vaccination cannot entirely eliminate pathogens from virus‐carrying animals [31]. FMD is a quintessential TAD with the potential to emerge sporadically in any disease‐free region. The recurrence of this disease is intricately linked to several factors, including the virus’s high adaptability and genetic diversity. Meanwhile, the virus’s high infectivity, rapid replication rate, and excretion from infected individuals significantly complicate epidemic control efforts [32]. Given that cattle are primarily affected by FMD, and considering that Inner Mongolia’s economy is largely centered around cattle farming, the prevention and control of FMD in Inner Mongolia have always been a top priority. Consequently, various regions within Inner Mongolia have implemented tailored prevention and control measures based on the prevailing epidemic situation, optimized vaccination protocols, raised livestock handlers’ awareness of animal diseases, and secured regional biosafety and disease‐free status, thereby safeguarding public health. In this study, bovine blood samples were collected from both households and farms across 8 districts of Tongliao City, Inner Mongolia Autonomous Region, China, between January 2021 and December 2022. The serum antibodies to bovine FMD O‐type and A‐type were detected.

### 4.1. Effects of Different Regions on the FMDV Antibody‐Positive Rates

In this study, 36,510 bovine blood samples were collected from various areas of Tongliao City between 2021 and 2022. The 2‐year test results revealed significant variation in positive rates, ranging from the minimum of 65.38% to the maximum of 100%. Significant differences were observed among the regions. From 2021 to 2022, the positive rates in Regions A and Regions E remained relatively stable. Compared with 2021, the positive rates in Regions B and Regions G significantly increased in 2022, indicating improved overall immune efficacy. However, the immune protection level of FMDV‐type antibodies in Regions B and G in 2021 was below 70%, suggesting a higher risk of viral infection. The literature indicates that the stability and elevated levels of FMD antibodies are closely linked to large‐scale vaccination programs. In endemic regions, maintaining an appropriate and consistent level of immunity is key to controlling and even eradicating FMD. Large‐scale vaccination can significantly increase antibody levels and duration of immunity, thereby effectively reducing the risk of disease transmission. However, factors such as vaccine stability, viral serotype diversity, and cold chain conditions can all influence antibody levels and the efficacy of immunity [33–35]. Therefore, it is imperative to enhance immunization efforts in these regions or implement supplementary immunization programs, along with effective preventive and control measures.

### 4.2. Effects of Different Sources on the FMDV Antibody‐Positive Rates

In this study, 16,921 and 19,589 cattle blood samples were collected from households and farms during 2021–2022, respectively. It revealed that the immunization effect of farms is higher than that of households. This finding aligns with prior research on immunoassays, which suggests that farming operations generally achieve higher immune efficacy compared to smaller, family‐run operations. For instance, in 2020, a serological survey in Tongliao City revealed that the positive rates of O‐type and A‐type FMDV immune antibodies in cattle from households were lower than those from farms [7]. In 2022, no significant difference was observed between farms and households. Consequently, it is imperative to enhance the vaccination efforts against FMD in households, including supplementary immunizations, adherence to immunization protocols, improved feed management, and routine disinfection practices, to effectively control and prevent the disease.

### 4.3. Effects of Different Seasons on the FMDV Antibody‐Positive Rates

Significant differences were observed among the seasons. In 2021, the positive rates of O‐type and A‐type antibodies were higher in spring and lower in autumn. In 2022, the positive rates of O‐type and A‐type antibodies were highest in winter and lowest in spring. Some studies have shown that the highest seroconversion rate was in the monsoon season (August to September). During this season, humidity and temperature increase, and the incidence of FMD cases also rises accordingly; in hot and humid seasons, the infection frequency of FMD is higher, which is due to the environment that is suitable for the persistence and spread of FMD, as the persistence of the airborne virus depends on high levels of moisture in the air [36–38]. These findings suggest that variations in antibody‐positive rates between years and seasons could be attributed to multiple factors, including vaccination programs, immunization protocols, feeding environments, daily management practices, biosecurity measures, and the technical proficiency of immunization procedures. From a management and biosecurity standpoint, the prevalence of conventional production systems with poor biosecurity infrastructure and inconsistent disinfection protocols among small‐ and medium‐sized livestock operations is a major contributor to pathogen introduction and spread, as well as reduced vaccine protective efficacy [39, 40]. European research data show that only 1% of pig farms in Europe conduct systematic evaluations of cleaning and disinfection effectiveness, and 40% of farms do not have boot disinfection equipment, indicating a significant gap in internal biosecurity implementation [41]. To address these deficits, strict enforcement of tiered disinfection regimens, zoned biocontainment controls, and standardized staff biosecurity training is required, complemented by routine environmental pathogen surveillance. These managerial interventions can mitigate disease outbreak risk and act synergistically with vaccination programs to improve the overall performance of comprehensive disease prevention and control.

### 4.4. Effects of Different Seasons and Sources on the FMDV Antibody‐Positive Rates

Significant differences were observed among the seasons and sources. The test results of this study demonstrate that the positive rates for spring, summer, autumn, and winter exceeded 80%. Specifically, the positive rates of FMDV O‐type and A‐type antibodies in households were lower in autumn and winter, and the positive rates in summer were significantly higher than those in winter. Differently, the positive rates of FMDV O‐type and A‐type antibodies in farms were lower in spring and autumn, and the positive rates in winter were significantly higher than in summer. These findings suggest that households should enhance their immunity during autumn and winter, whereas farms should focus on strengthening immunity in spring and autumn. This indicates that there are distinct seasonal patterns of immunization between households and cattle farms, which may lead to varying immunization outcomes. Chanchaidechachai et al. conducted a statistical analysis of the outbreak rates of FMD in three different seasons (rainy season, winter, and summer) in Thailand’s FMD outbreak reports from 2011 to 2018. Seasonal factors indicated that the outbreak rate was lower from February to June, then rose and reached its peak from October to December, and thus, winter was the outbreak season of FMD [42]. Pedro Mil‐Homens et al. conducted a statistical analysis of reported outbreaks of FMD in domesticated animals in Turkey from 2005 to 2025. The seasonal pattern showed that the outbreak concentration was higher in spring [43]. Therefore, it is essential to intensify monitoring efforts for both households and farms, develop optimized immunization programs tailored to the seasonal differences, and implement supplementary immunization measures as appropriate to minimize the risk of viral infections.

### 4.5. Strengthen Vaccine Effects on FMD Prevention and Control

Compulsory vaccination of cattle farms is a critical measure for preventing and controlling FMD. In accordance with the National Guidelines on Compulsory Immunization against Animal Diseases (2022–2025), the recommended immunization procedures are as follows. For large‐scale farms, vaccination timing is determined based on maternal antibody levels: Piglets receive primary vaccination at 28–60 days old, lambs at 28–35 days old, and calves at approximately 90 days old. Following the initial vaccination, all neonatal livestock should receive a booster dose 1 month later, followed by subsequent vaccinations every 4–6 months. For households, all susceptible animals should be vaccinated in both spring and autumn, with monthly supplementary vaccinations as needed. Where feasible, farms can follow the immunization schedules specific to large‐scale operations. Meanwhile, in the event of an outbreak, emergency vaccinations should be administered to farms in affected and threatened areas based on emergency monitoring or risk assessment. Additionally, if border regions are at risk due to overseas outbreaks, farms in high‐risk areas should undergo emergency vaccination according to the results of risk assessments. Hence, regular serum antibody monitoring for FMD O‐type and A‐type immunization efficacy can reduce FMDV outbreak probability, mitigate livestock economic losses, and advance sustainable development of the breeding industry. It is widely acknowledged that administering a vaccine earlier, prior to an outbreak, significantly enhances its protective efficacy [44]. However, the FMD vaccine offers limited cross‐protection against various viral strains and does not induce a fully sterile immune response. Consequently, regular booster vaccinations are required to sustain the vaccine’s protective potency [45]. In historically FMD‐free countries, maintaining disease‐free status necessitates routine vaccination, and vaccination‐based FMD freedom is deemed a preferred national disease‐control strategy for many nations [46]. Furthermore, numerous factors can influence vaccine effectiveness, including improper immunization operations, insufficient epidemic prevention measures, substandard vaccine usage, flawed immunization protocols, and inadequate transportation and storage conditions, all of which may compromise vaccine performance and diminish immunization effects. Therefore, it is imperative for farms to ensure the proper sterilization of vaccine syringes, needles, and injection methods, optimize and adjust immunization procedures, and adhere to strict guidelines for vaccine transportation, storage, and administration. In summary, refining the immunization strategy and closely monitoring the immunization status of FMD can facilitate timely supplementation and exemption, standardize vaccine use, and contribute to enhancing the health of livestock and ensuring public health safety.

This large‐scale serological survey served as routine regional epidemiological monitoring for FMD in Tongliao City, yet several limitations exist. In total, 36,510 blood samples were gathered from smallholder households and dispersed farms covering eight administrative divisions. Practical field constraints prevented complete standardized individual animal records (species, age, sex, exact days postvaccination) for all sampled livestock, a typical difficulty for wide‐scale cross‐regional serosurveillance of rural free‐range cattle herds. Large sample quantities and limited on‐site diagnostic capacity precluded NSP antibody testing, which prevents distinguishing vaccinated cattle from naturally infected animals. The detected positive antibodies in this study mainly reflected the overall immune status of the cattle herds under compulsory immunization programs, rather than pure vaccine efficacy.

## 5. Conclusions

In this study, 36,510 bovine blood samples were tested using a liquid‐phase blockade ELISA method. The test results were analyzed across different regions, sources, seasons, and years. A significant difference was observed among the regions, sources, seasons, and years. The positive rates of FMDV O‐type and A‐type antibodies exceeded 70% in most regions, with the exception of Regions B and G, where the positive rates of FMDV A‐type antibodies were below 70%. The immunization effect was generally satisfactory, meeting the requirements outlined in the National Guidelines on Compulsory Immunization against Animal Diseases (2022–2025). Regions that did not meet these standards should enhance their vaccination efforts, ensuring thorough catch‐up vaccinations and reevaluations. Currently, FMD poses a significant threat to the cattle breeding industry, causing substantial economic losses. Therefore, both households and farms must strictly follow the standardized vaccine usage guidelines to minimize factors that may affect vaccine efficacy; through regular serological monitoring, they can dynamically grasp the antibody levels of herd immunity, scientifically assess the immunization effect, and optimize the vaccination schedule. At the same time, molecular monitoring of the FMDV can be conducted based on field isolates to provide technical support for the formulation of regional vaccine immunization strategies, tracing of transmission, and epidemic prevention and control. Supplementary immunization and comprehensive prevention measures can also be implemented to enhance the overall health level of the cattle herd and effectively reduce the risk of FMD outbreaks.

## Author Contributions

Rong Zhao: methodology, data curation, and writing–original draft; Xiong Dai: formal analysis and resources; Jun Ai: data curation, supervision, project administration, and funding acquisition; Tianlong Hu: investigation; Ruiying Wang: visualization; Yanlin Li: investigation and software; Jige Xin: conception, supervision, funding acquisition, and writing–original draft; Diangang Han: investigation, funding acquisition, and writing–review and editing.

## Funding

This research was supported by the Expert Workstation of Yunnan (202505AF350101), the National Key Research and Development Program of China (2022YFC2601605), the Yunnan Key Laboratory of Veterinary Etiological Biology (202449CE340019), and the Program of Yunnan High‐level Talents to Young Talents (YNWR‐QNBJ‐2020‐154).

## Ethics Statement

The animal study protocol was approved by the Ethics Committee of Life Sciences at Yunnan Agricultural University (protocol code No. 202012001, 12/31/2020).

## Consent

The authors have nothing to report.

## Conflicts of Interest

The authors declare no conflicts of interest.

## Data Availability

Data are available upon request from the authors.
